# Evaluation of Change in Canine Diagnosis Protocol Adopted by the Visceral Leishmaniasis Control Program in Brazil and a New Proposal for Diagnosis

**DOI:** 10.1371/journal.pone.0091009

**Published:** 2014-03-07

**Authors:** Wendel Coura-Vital, Henrique Gama Ker, Bruno Mendes Roatt, Rodrigo Dian Oliveira Aguiar-Soares, Gleisiane Gomes de Almeida Leal, Nádia das Dores Moreira, Laser Antônio Machado Oliveira, Evandro Marques de Menezes Machado, Maria Helena Franco Morais, Rodrigo Corrêa-Oliveira, Mariângela Carneiro, Alexandre Barbosa Reis

**Affiliations:** 1 Laboratório de Pesquisas Clínicas, Ciências Farmacêuticas, Escola de Farmácia, Universidade Federal de Ouro Preto, Morro do Cruzeiro, Ouro Preto, Minas Gerais, Brazil; 2 Infectologia e Medicina Tropical, Faculdade de Medicina, Universidade Federal de Minas Gerais, Minas Gerais, Brazil; 3 Laboratório de Imunopatologia, Núcleo de Pesquisas em Ciências Biológicas, Instituto de Ciências Exatas e Biológicas, Universidade Federal de Ouro Preto, Morro do Cruzeiro, Ouro Preto, Minas Gerais, Brazil; 4 Secretaria Municipal de Saúde, Prefeitura de Belo Horizonte, Belo Horizonte, Minas Gerais, Brazil; 5 Laboratório de Imunologia Celular e Molecular, Centro de Pesquisas René Rachou, Fundação Oswaldo Cruz, Belo Horizonte, Minas Gerais, Brazil; 6 Laboratório de Epidemiologia de Doenças Infecciosas e Parasitárias, Departamento de Parasitologia, Instituto de Ciências Biológicas, Universidade Federal de Minas Gerais, Belo Horizonte, Minas Gerais, Brazil; 7 Instituto Nacional de Ciência e Tecnologia em Doenças Tropicais (INCTDT), Salvador, Bahia, Brazil; University of São Paulo, Brazil

## Abstract

The techniques used for diagnosis of canine visceral leishmaniasis (CVL) in Brazil ELISA and IFAT have been extensively questioned because of the accuracy of these tests. A recent change in the diagnosis protocol excluded IFAT and included the Dual-Path Platform (DPP)_._ We evaluated the prevalence and incidence rates of *Leishmania* spp. before and after the change in the protocol. In addition, based on our results, we propose a new alternative that is less expensive for the screening and confirmation of CVL. Plasma samples were obtained from a serobank from dogs evaluated in a cross-sectional study (1,226 dogs) and in a cohort study of susceptible animals (n = 447), followed for 26 months. Serology testing was performed using ELISA, IFAT, and DPP. The incidence and prevalence of CVL were determined by using the protocol of the Visceral Leishmaniasis Control and Surveillance Program until 2012 (ELISA and IFAT using filter paper) and the protocol used after 2012 (DPP and ELISA using plasma). The prevalence was 6.2% and the incidence was 2.8 per 1,000 dog-months for the protocol used until 2012. For the new diagnosis protocol for CVL resulted in an incidence of 5.4 per 1,000 dog-months and a prevalence of 8.1%. Our results showed that the prevalence and incidence of infection were far greater than suggested by the previously used protocol and that the magnitude of infection in endemic areas has been underestimated. As tests are performed sequentially and euthanasia of dogs is carried out when the serological results are positive in both tests, the sequence does not affect the number of animals to be eliminated by the Control Program. Then we suggest to municipalities with a large demand of exams to use ELISA for screening and DPP for confirmation, since this allows easier performance and reduced cost.

## Introduction

In recent decades visceral leishmaniasis (VL) has become a major public health problem in Brazil, affecting approximately 3,379 individuals per year, with an annual incidence rate of 1.9 cases per 100,000 [1]. Zoonotic VL is a potentially fatal disease that is transmitted by the Phlebotominae sandfly species and is caused by the intracellular protozoan parasite *Leishmania infantum* (*Leishmania chagasi*), which is endemic in South America, Central America, the Mediterranean basin, and parts of Asia [2].

Dogs are highly susceptible to leishmaniasis infection and represent the major source of infection because of intense skin parasitism, independent of their clinical presentation [3]–[5]. The domestic dog has been identified as the main urban reservoir for *L. infantum*, and canine VL (CVL) is considered to be an emerging and re-emerging disease, as indicated by an increase in the number of seropositive dogs and by the geographical expansion of the disease [6]–[10].

To control the spread of disease, the Brazilian Ministry of Health through the Visceral Leishmaniasis Control and Surveillance Program (VLCSP) has instituted various measures including early diagnosis and treatment of human cases, identification and culling of seropositive infected dogs, control of insect vectors, and health education [1]. The VLCSP mainly relies on the euthanasia of seropositive dogs to control VL; however, this measure is controversial and some reports suggest that it has little impact on the reduction of human and canine cases [11]–[13]. This failure has been attributed to delays in detecting and eliminating infected dogs, the tendency to replace infected dogs with susceptible puppies, and the low sensitivity of the serological methods used [14]–[19]. Some studies have estimated that the sensitivity of the immunofluorescent antibody test (IFAT) ranges from 68% to 100% and that the specificity ranges from 52% to 100%, whereas the sensitivity of enzyme-linked immunosorbent assay (ELISA) ranges from 91% to 97% and the specificity of ELISA ranges from 83% to 98% [16], [20]–[23].

Until 2012, CVL had been diagnosed using IFAT, a method recommended for confirming positive cases detected by ELISA. These serological tests were performed using serum or blood samples collected on filter paper [1], [19]. Recently, to improve accuracy in the diagnosis of CVL, the VLCSP has recommended using an immunochromatographic rapid test comprising rK26 and rK39 recombinant antigens, the Dual-Path Platform (DPP; Bio-Manguinhos/Fiocruz, Rio de Janeiro, Brazil), for the screening of infected dogs and ELISA to confirm the positive results [24]–[25]. Moreover, changing the eluate from dried blood collected on filter paper to serum or plasma samples has been recommended [24]. However, this measure has not yet been widely adopted by all health departments of the municipalities located in many endemic areas around the country because of some operational difficulties.

Herein, we report the results of a baseline canine survey followed by a cohort study using the conventional serological methods used by the VLCSP until 2012 (ELISA and IFAT with filter paper) and after 2012 (DPP and ELISA with plasma) to evaluate the prevalence and incidence of *Leishmania* spp. infection in a large canine population in an endemic area of Brazil. In addition, based on our results, we propose a new alternative that is less expensive for the screening and confirmation of CVL.

## Methods

### Ethical Statement

The study was approved by the Committees of Ethics in Animal Experimentation of the Universidade Federal de Ouro Preto (protocol no. 083/2007), of the Universidade Federal de Minas Gerais (protocol no. 020/2007), and of the City Council of Belo Horizonte (protocol no. 001/2008). All procedures in this study were according to the guidelines set by the Brazilian Animal Experimental Collage (COBEA), Federal Law number 11794. Owners of the dogs participating in the project were informed of the research objectives and were required to sign the Informed Consent Form before sample collection.

### Study Design: Initial Survey and Follow-up

Plasma samples were provided by the serobank of the Clinical Research Laboratory of the School of Pharmacy at the Federal University of Ouro Preto and were selected from a baseline survey and prospective cohort study performed from June 2008 to August 2010 in Belo Horizonte, Minas Gerais State, Brazil [7], [10]. Briefly, a cross-sectional study was performed in the northwest sanitary district (36,874 km^2^) of Belo Horizonte. According to zoonosis control management, the canine population comprised 20,883 dogs. With an expected positive rate of 5% to 10% for CVL in the study area 95% confidence interval (CI) and an estimated precision rate of 1.5%, the appropriate sample size for the study was 1,226 dogs. The field work was performed in close collaboration with the Municipal Health Service, and the data were collected during the canine survey census conducted by health agents as a routine procedure of the VLCSP.

The follow-up study was initiated 10 months after the baseline (evaluation I) with 447 dogs and blood samples were collected by venipuncture. Evaluations II and III were conducted 16 and 26 months after the baseline, respectively. All dogs included in evaluations II and III underwent the same procedures as used in evaluation I.

**Figure 1:**
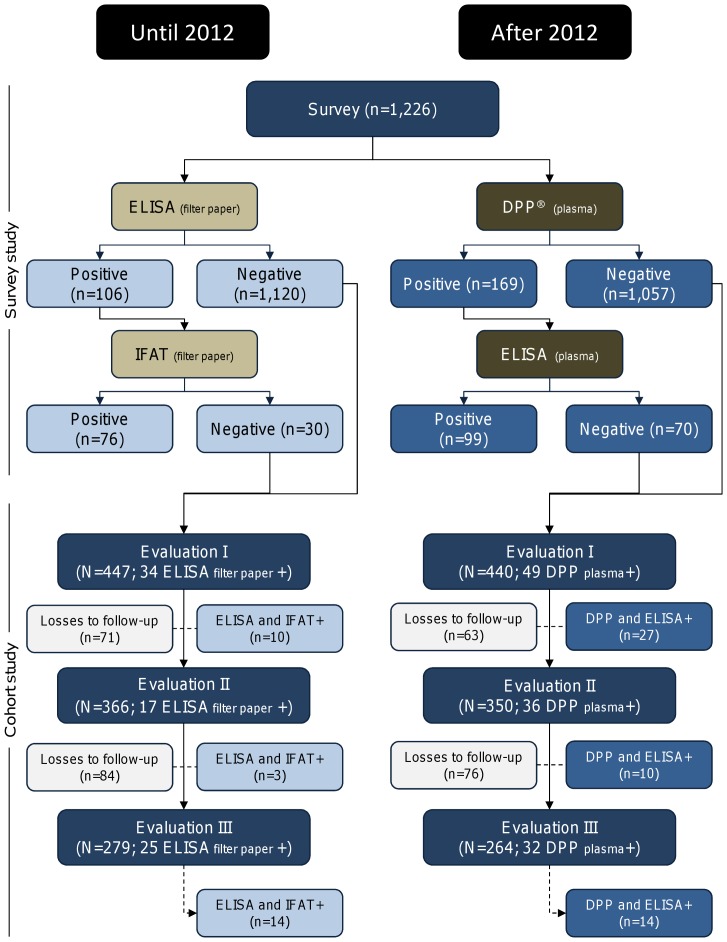
Diagnostic protocols used for canine visceral leishmaniasis in Brazil until 2012 and after 2012.

**Figure 2:**
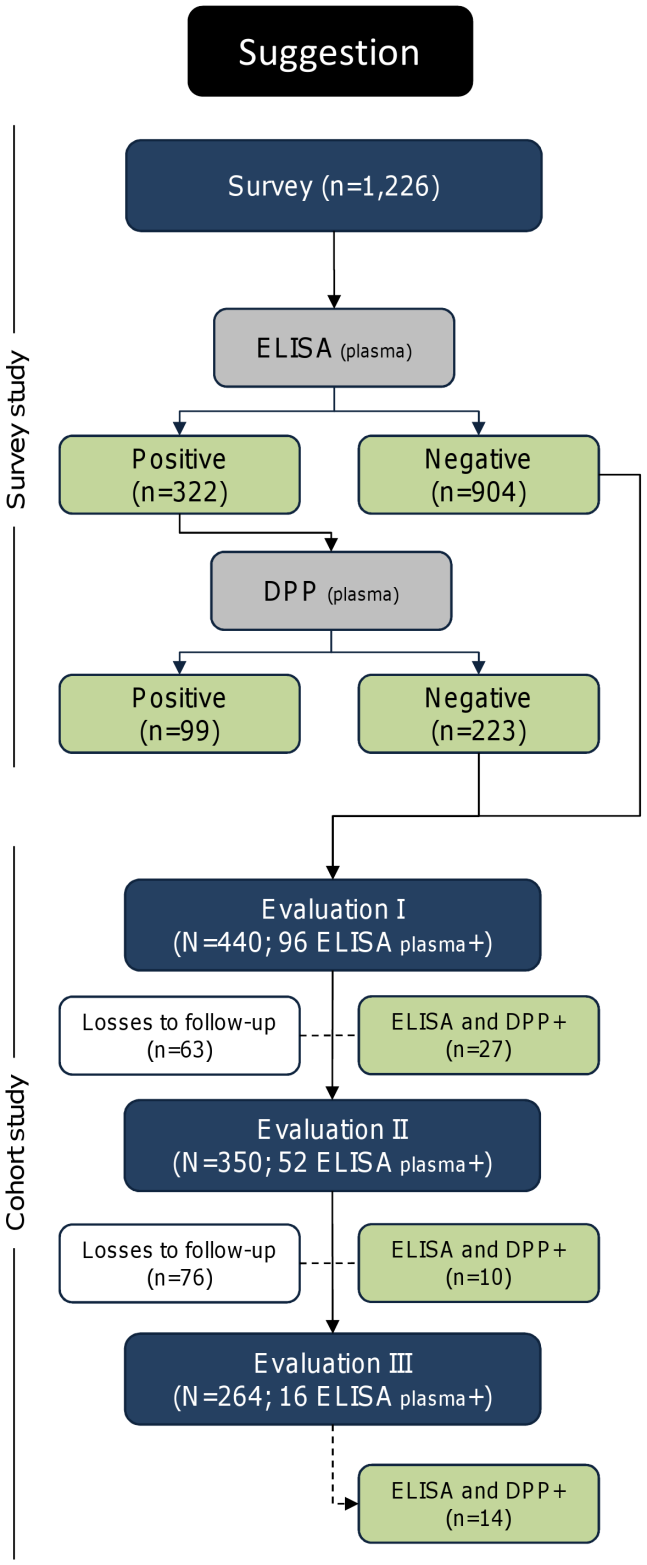
Study design using a new proposed method for screening and confirmation of canine visceral leishmaniasis.

During the follow-up, losses occurred due to seropositivity in sequential tests, death, change of address, household closed, refusal, and dog escape (Fig. 1 and 2). The number of losses during follow-up in each evaluation changed according to the protocol employed in the diagnosis.

### Collection of Blood Samples

A sample of peripheral blood (5 mL) was collected by puncture of the brachiocephalic vein, and an aliquot was transferred to a glass vial containing sufficient anticoagulant (ethylenediaminetetraacetic acid) to achieve a final concentration of 1 mg/mL. Then the blood sample was transferred from the syringe to filter paper and maintained at room temperature until dry out. The filter paper sample was then sent to the Laboratory of Zoonosis of the Municipality of Belo Horizonte and analyzed. The blood sample was centrifuged (1,500–1,800 *g*, 20 min), and the plasma was collected and stored at –70°C from 3 to 5 years until it was assayed for the serological tests (DPP and ELISA) in the Laboratory of Immunopathology of the Federal University of Ouro Preto. Serological tests conducted in the Laboratory of Immunopathology were performed blind.

### Serological Tests (ELISA, IFAT, and DPP)

Each sample was tested using two protocols established by the Brazilian Ministry of Health and a third protocol proposed by our group. In all protocols the tests were performed sequentially. The first used ELISA (Canine Leishmaniasis EIE Kit; Bio-Manguinhos/Fiocruz) for screening and IFAT (Canine Leishmaniasis IFI Kit; Bio-Manguinhos/Fiocruz) as a confirmatory test. This protocol used blood collected on filter paper (eluate) to perform the serological tests. The second protocol used the DPP CVL rapid test (Bio-Manguinhos/Fiocruz) for screening and ELISA (Canine Leishmaniasis EIE Kit) as a confirmatory test. This protocol used plasma for the serological tests. Both protocols followed the manufacturer’s instructions. As an alternative, the third protocol used ELISA (Canine Leishmaniasis EIE Kit) for screening and DPP CVL rapid test for confirmation. The cut-off of the EIE Kit was defined based on the manufacturer’s instructions and by considering the mean of the optical density of the negative controls multiplied by two. IFAT to detect anti-*Leishmania* IgG antibodies was performed as described by Camargo [26] using the Canine Leishmaniasis IFI Kit. Dogs with antibody titrations equal or higher than 1∶40 were considered to be positive for disease.

### Statistical Analysis

Statistical analysis was performed using Stata software (version 11.0; Stata Corp, College Station, TX). The prevalence and incidence rates indicated by ELISA, IFAT, and DPP were estimated using 95% CI.

## Results

### Serological Baseline Survey

Among the 1,226 dogs, 106, 169, and 322 dogs had seropositive results according to ELISA using filter paper (ELISA-FP), DPP, and ELISA using plasma (ELISA-PL), with estimated prevalence rates of 8.6% (95% CI, 7.1–10.4), 13.8% (95% CI, 11.8–16.0), and 26.3% (95% CI, 23.8–28.8), respectively. Among the ELISA-FP–positive results (106), 76 (71.7%) were confirmed by IFAT. Independent of the protocol of diagnostic test used in screening or confirmatory phase (DPP or ELISA-PL), the same number of dogs (99) was found positive for infection in both tests (Figs. 1 and 2). After the confirmatory tests (sequential), the prevalence rates were 6.2% (95% CI, 4.9–7.7) for the first protocol (ELISA-FP and IFAT), 8.1% (95% CI, 6.6–9.8) for the second protocol (DPP and ELISA-PL), and 8.1% (95% CI, 6.6–9.8) for the third protocol (ELISA-PL and DPP) (Table 1).

**Table 1:** Estimated prevalence of canine visceral leishmaniasis using two strategies of serological sequential testing.

Diagnostics Methods	Prevalence (95% CI)
ELISA and IFAT (filter paper)	6.2 (4.9–7.7)
DPP and ELISA (plasma)	8.1 (6.6–9.8)

CI, confidence interval; DPP, Dual-Path Platform; ELISA, enzyme-linked immunosorbent assay; IFAT, immunofluorescent antibody test.

### Incidence of Seroconversion using Sequential Testing

According to ELISA-FP and IFAT, 27 seroconversions were observed in both tests within the study cohort, with an overall incidence rate of 2.8 per 1,000 dog-months (95% CI, 1.9–4.1). However, for DPP and ELISA-PL 51 seroconversions were observed, with an incidence rate of 5.4 per 1,000 dog-months (95% CI, 4.1–7.1) (Table 2).

**Table 2:** Dog-months of follow-up, seroconversion in sequential testing, and incidence rates in Brazil.

	Diagnostic Methods			
Follow-up	ELISA and IFAT (Filter Paper)		DPP and ELISA (Plasma)	
	Seroconversion	Incidence Rate^d^(95% CI)	Seroconversion	Incidence Rate^d^(95% CI)
Evaluation I^a^	10	1.9 (1.0–3.5)	27	5.1 (3.5–7.4)
Evaluation II^b^	3	1.4 (0.5–4.5)	10	5.0 (2.7–9.3)
Evaluation III^c^	14	6.2 (3.6–10.4)	14	6.4 (3.8–10.8)
Total	27	2.8 (1.9–4.1)	51	5.4 (4.1–7.1)

a At 10 months after baseline.

b At 16 months after baseline.

c At 26 months after baseline.

d Incidence rate per 1,000 dog-months. CI, confidence interval; DPP, Dual-Path Platform; ELISA, enzyme-linked immunosorbent assay; IFAT, immunofluorescent antibody test.

## Discussion

In the present study we compared, for the first time, the change of the protocols for the diagnosis of CVL in Brazil. We performed a baseline survey using 1,226 dogs, followed by a cohort study using 447 dogs. Our results showed that the protocol using the DPP and ELISA detected a higher prevalence (8.1%) and incidence (5.4/1,000 dog-months) of infected dogs than did the protocol using ELISA and IFAT (prevalence, 6.2%; incidence, 2.8/1,000 dog-months). Previous studies showed that DPP had good performance, with sensibility ranging from 93% to 100% and specificity ranging from 92% to 100% [25], [27]–[29]. However, Grimaldi et al. [25] observed that the sensitivity depended on the clinical status, which was higher in symptomatic than in asymptomatic dogs. In the present study, the majority of dogs evaluated were classified as asymptomatic [7], [10]; however, DPP and ELISA still showed better performance. It is important to note that the sensitivity of a diagnostic test changes during the clinical course of infection [30]–[31]. Quinnell et al. [33] evaluated the diagnostic performance of immunochromatographic dipstick RDTs using rK39 antigen for CVL and showed that antibody responses to rK39 in natural infection develop slower than do responses to crude antigen. However, as observed in the present study, the combination of recombinant antigens rK39 and rK26, as in DPP, can improve this performance.

IFAT is the most widespread diagnostic method. For many years, IFAT was considered the serological reference test for CVL. However, this technique presented limitations such as low reproducibility, especially when filter paper was used, higher false-positive results because of cross-reactivity with *Trypanosoma cruzi*, *Trypanosoma caninum*, *Leishmania braziliensis*, and *Ehrlichia canis*, and false-negative results attributable to other factors [19]–[20], [29]. Moreover, IFAT may be impractical for use in rural areas because of the lack of laboratory equipment and qualified technicians to interpret the results. Although ELISA also presented cross-reactivity, its sensitivity, specificity, and reproducibility were better than that of IFAT [20], [28]. Furthermore, ELISA is an automated technique that enables the testing of a large number of samples simultaneously and is less subjective than IFAT. During the evaluation of serological cross-reactivity between CVL and *T. caninum*, Alves et al. [29] observed that a large percentage of dogs were erroneously diagnosed with CVL even after screening by ELISA and confirmation by IFAT. When evaluating the DPP test, this authors observed minor cross-reactivity [29]. Use of recombinant proteins, such as rK39 and rK26, has led to excellent results in the detection of human VL and CVL, independent of the diagnostic method used [28], [31]–[34]. Additionally to the aspects mentioned above, the best results obtained with the new protocol are also associated to the quality of the samples analyzed; considering the higher sensitivity in plasma than when filter paper is used [15], [35].

An improvement in the accuracy of the tests used for the diagnosis of CVL is critical for the VLCSP [18]. Considering the new protocol for CVL diagnosis and the results obtained in the present study, we believe it is important to discuss the factors related to the viability of using DPP in accordance with the logistics of the VLCSP.

The DPP has advantages such as easy storage, rapidity as a diagnostic method, ease of use, and flexibility in the type of biological samples used (blood, serum, or plasma) [24]–[25]. However, although the test can be used in the field with a simple visual assessment of positivity, the use of an optical reader increases the credibility of the results, as previously recommended by Grimaldi et al. [25]. Furthermore, the use of DPP in the field, in municipalities with a large number of cases, leads to a reduction in the number of dogs monitored daily by health agents, because it is necessary to collect samples, perform the assay in the home, and subsequently performs new blood collection in seropositive dogs for confirmation by ELISA. Another option is to perform the sample collection in seropositive dogs later; however, returning home with dogs that are declared seropositive by DPP results in a great loss of time and a loss of dogs, making this option impracticable in large municipalities or in large-scale surveys. With the first option, it is necessary to establish a daily routine of sending the blood samples to the laboratory for storage and processing. Because the logistics and structure of sending and processing are the same for large or small numbers of samples, collecting samples from all dogs and using diagnostic tests in the laboratory are better options. In this context, in many Brazilian states the recommendation is that blood collection in the field is carried out in all animals and sent to the Zoonosis Control Centers for DPP testing. Next, the positive samples are sent to the Central Public Health Laboratories (LACENS). This eliminates the need to maintain a structured laboratory for performing ELISA in small municipalities. By using this methodology the Brazilian Ministry of Health reduces the demand for tests performed (ELISA) in the LACENS.

However, based on the current guidelines of the Ministry of Health and the data obtained in this study, we suggest to municipalities with a large demand of exams to use ELISA for automated screening and DPP as confirmatory test. This strategy would allow diagnosis in a laboratory where quality control can be easily implemented, thus facilitating the performance of large-scale screening tests. In addition, the cost of the ELISA reaction is lower (US $1.63) than the cost of the DPP test (US $2.72), when performed on large scale. Moreover, these values are related to the price of the tests only and do not include the transportation of inputs, acquisition and maintenance of equipment or training of specialized technicians.

All these methods need to be significantly improved because the results determine whether seropositive dogs in endemic areas will be euthanized. In addition to the direct consequence experienced by the dogs (euthanization), there are also consequences for the dog owners, such as emotional trauma. Furthermore, false-negative results are unacceptable because they may lead to the perpetuation of infection. A possible way to improve canine diagnosis is the use of ELISA with recombinant antigen, such as rK39 and rK26, since they exhibit excellent performance [15], [34], [36].

Important features of this study must be highlighted. The analysis assays were carried out with samples obtained in the epidemiological studies (i.e., cross-sectional and concurrent cohort) to evaluate prevalence and incidence in a large number of dogs evaluated in an urban endemic area.

To avoid diagnosis bias, the assays were performed in blind (DPP and ELISA). Also, we took care not to identify sample results according to previous exams of ELISA and IFAT carried out in filter paper.

In conclusion, the new protocol for diagnosis of CVL (DPP and ELISA) shows that the prevalence and incidence of infection are far greater than suggested by the previous protocol (ELISA and IFAT), indicating that the magnitude of infection in endemic areas has been underestimated. As tests are performed sequentially and euthanasia of the dog is carried out when the serological results are positive in both tests, the sequence does not affect the number of animals to be eliminated by the Control Program. Then, we suggest to municipalities with a large demand of exams to use ELISA for screening and DPP for confirmation because of its easy use and reduced costs.
